# A systematic review on the level of risk perception of diabetes mellitus: The role of environmental factor

**DOI:** 10.1371/journal.pone.0308152

**Published:** 2024-07-30

**Authors:** Miaw Yn Jane Ling, Norfazilah Ahmad, Azimatun Noor Aizuddin, Mohd Hasni Ja’afar

**Affiliations:** Department of Public Health Medicine, Faculty of Medicine, Universiti Kebangsaan Malaysia, Cheras, Kuala Lumpur, Malaysia; Xiamen University - Malaysia Campus: Xiamen University - Malaysia, MALAYSIA

## Abstract

**Background:**

Risk perception plays important role in motivating preventive health behaviours. The objective of this systematic review was to explore the level of diabetes risk perception among individuals with and without apparent risk for diabetes, and to consider the effect of environmental factors on the level of diabetes risk perception.

**Methods:**

This systematic review was reported according to the Preferred Reporting Items for Systematic Reviews and Meta-Analyses guidelines. The literature search was carried out through PubMed, Web of Science, and Scopus. Original articles written in English and published between 2013 and 2023 were considered. Study quality was appraised using the Mixed Methods Appraisal Tool. Narrative synthesis was undertaken due to methodological heterogeneity in the included studies.

**Results:**

A total of 13 cross-sectional studies, two randomized controlled trials, two cohort studies, two mixed methods studies and one quasi-experiment with a control group were included. An overall low level of diabetes risk perception was reported particularly in those without apparent risk for diabetes. The 20 included studies reported widely varied measures for calculating diabetes risk perception. The influence of environmental factors on the risk perception of diabetes was highlighted.

**Limitations:**

The use of study-specific and non-validated measures in the included studies weakens the authors’ ability to compare across studies. The role of language and publication bias within this systematic review should be acknowledged as we included only English-language studies published in peer-reviewed journals. Another limitation is the exclusion of dimensions of risk perception such as optimistic bias as search terms.

**Conclusion:**

The overall low risk perception of diabetes calls for urgent need of public health interventions to increase the risk perception of diabetes. In the future, researchers should ensure the validity and reliability of the measures being used. The influence of environmental factors on the diabetes risk perception indicates that diabetes preventive interventions targeting environmental factors may be effective in increasing the risk perception of diabetes.

## Introduction

Diabetes is a chronic disease which occurs due to the insufficient production of insulin by the pancreas or due to the inability of the body to use insulin effectively. Majority (95%) of people with diabetes were diagnosed with type 2 diabetes, which most often develops in adults [1]. Globally, an estimated 537 million people have diabetes in 2021 [2], which has sharply increased compared to 108 million in 1980 [3]. Both the incidence and prevalence of diabetes have increased markedly in all low and high income regions, which are attributable to the rise in diabetes risk factors like being overweight or obese [3, 4].

The increasing trends of mortality and disability-adjusted life-years (DALYs) associated with diabetes from 1990 to 2017 have been observed. The age-standardized mortality rate due to diabetes increased from 15.7 to 17.5, while age-standardized DALYs increased from 717.7 to 839.0 [4]. Regarding economic impact, the estimated global health expenditure on diabetes was US$760 billion in 2019. This figure is expected to increase to US$845 billion by 2045 [5]. Additionally, diabetes was found to have a significant negative impact on the ability to work which places a considerable economic burden on the society [6].

The World Health Organization stated that the four main modifiable behavioural risk factors contributing to non-communicable diseases (NCDs) such as diabetes include unhealthy diet, harmful use of alcohol, tobacco use and physical inactivity [7]. According to the Health Belief Model (HBM), health-related behaviours are influenced by risk perception [8], which refers to people’s subjective judgement about the likelihood of negative events, including injury, illness, disease and death [9]. The HBM posits that, for a person to change his/her behaviours, he/she needs to perceive the likelihood of developing a disease, concern about the severity of the disease, and perceives the benefit of taking action towards preventing the diseases taking into consideration the perceived cost or barrier [8].

In line with the HBM, many studies found that an individual’s perception of developing a disease is an important factor towards adopting healthy lifestyles and the uptake of preventive interventions. For example, one study found that cardiovascular disease patients with higher risk perception of having cardiovascular disease again in the future had higher likelihood of adhering to medications and attending cardiac rehabilitation [10]. In other studies, those who had high risk perception of breast cancer were more likely to undergo mammography examination [11], while those who had high risk perception of diabetes were more likely to participate in sufficient physical activity [12].

Reflecting the importance of risk perception in influencing health-related behaviours, many studies have been conducted to assess the level of risk perception of NCDs including diabetes [13, 14]. The level of risk perception from these studies varies, probably due to difference in study population and tools used to measure the risk perception of diabetes. In view of the rising burden of diabetes, it is imperative to have a better understanding of its risk perception as a guide for the development of preventive interventions. A systematic review will help to fill the research gaps in the knowledge of risk perception of diabetes. Nevertheless, to date, systematic review on the level of risk perception of diabetes is limited. A previous systematic review on the risk perception of diabetes has focused only on Asian Americans who are at increased risk of diabetes compared with non-Hispanic Whites [15]. The lack of ethnic diversity represents an important research gap in this research area that should be addressed.

Therefore, the objective of this systematic review was to explore the level of risk perception of diabetes among individuals with and without apparent risk for diabetes. Apart from that, the present systematic review considers the role of environmental factors that may influence the level of diabetes risk perception. While realizing the importance of environmental factors, this systematic review focuses on the environmental factors through a general review of the scientific literature on the subject.

## Materials and methods

This systematic review was carried out according to the Preferred Reporting Items for Systematic Reviews and Meta-Analyses (PRISMA) 2020 statement [16] (S1 Appendix). The protocol of this systematic review was registered in PROSPERO (registration ID: CRD42023390752).

### Research question formulation

The review question was developed based on the PICO (population, intervention, comparison, outcome) format. The PICO format may be used for etiology questions that ask to what extend a certain factor or condition is highly associated with an outcome [17]. The question was structured as follows: Do individuals (population) who had apparent risk for diabetes (intervention) compared with individuals without apparent risk for diabetes (comparison) perceive higher level of risk of diabetes mellitus (outcome)?

### Data source and search strategy

**Fig 1:**
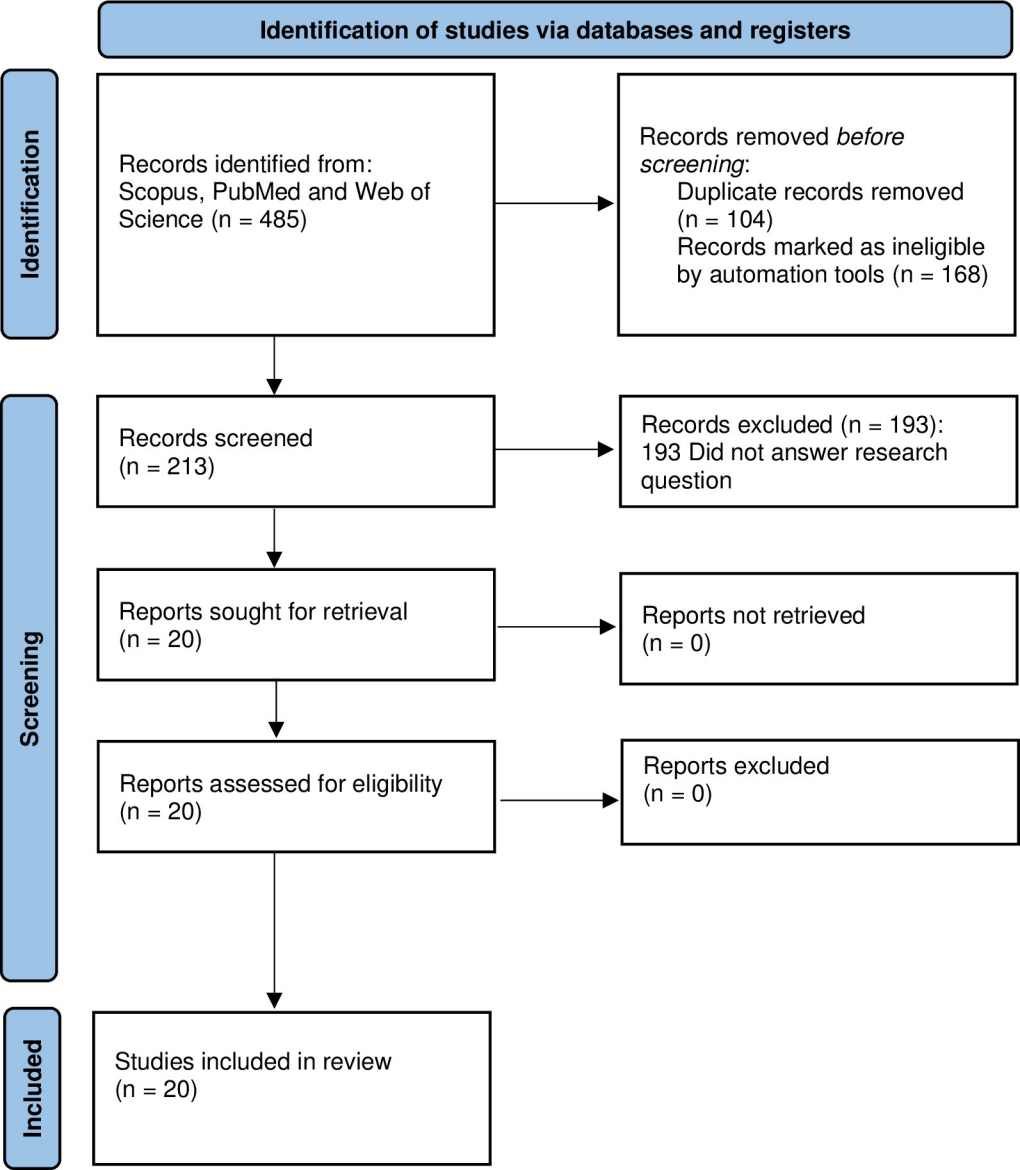
PRISMA flow diagram.

A total of three databases including PubMed, Web of Science and Scopus were searched on 23 January 2023. The keywords used for the search of related articles are provided in Table 1. There were 485 records identified from the three databases, including 295 records from Scopus, 106 records from Web of Science and 84 records from PubMed. Automated tools were used and 168 records were excluded based on publication type (article), language (English), and publication year (2013–2023). A total of 104 duplicate records were found and removed, leaving 213 records for title screening (Fig 1). The records were exported from the databases into an Excel sheet for screening.

**Table 1:** Keywords used in the screening process.

Database	Search string
Scopus	TITLE-ABS-KEY(("degree" OR "level") AND ("risk perception" OR "perceived risk") AND ("diabetes" OR "diabetes mellitus" OR "type 2 diabetes" OR “type 2 diabetes mellitus”))
Web of Science	TS = (("degree" OR "level") AND ("risk perception" OR "perceived risk") AND ("diabetes" OR "diabetes mellitus" OR "type 2 diabetes" OR “type 2 diabetes mellitus”))
PubMed	((degree[Title/Abstract] OR level[Title/Abstract]) AND (risk perception[Title/Abstract] OR perceived risk[Title/Abstract])) AND (diabetes[Title/Abstract] OR diabetes mellitus[Title/Abstract] OR type 2 diabetes[Title/Abstract] OR type 2 diabetes mellitus[Title/Abstract])

### Inclusion and exclusion criteria

The inclusion criteria were: (1) publication in English language; (2) original article; (3) publication between 2013–2023. Articles that included information on the level of risk perception of diabetes were included, while non-original articles such as conference proceedings, commentary, reports, review articles and systematic reviews were excluded.

### Study selection

The titles and abstracts were independently screened by three reviewers (MYJL, NA, and ANA) according to the review question. Of the 213 articles, 20 were identified as potentially eligible articles through screening by titles and abstracts. A total of 193 articles were excluded, including: i) 67 articles that reported the results of risk perception other than that of diabetes, ii) 50 articles that were related to diabetes but not to risk perception, iii) 36 articles that were related to the risk perception of diabetes but did not report its level [i.e. qualitative studies, studies that measure risk perception as dichotomous (yes or no) response, studies that reported the results of risk perception of NCDs in general], iv) 26 articles that were not related to diabetes or risk perception, v) 7 articles that were review articles, and vi) 7 articles that were protocols with no results.

Full text were retrieved for the 20 articles for assessment of eligibility. Disagreements were resolved through discussion and consensus among the three authors and input from a fourth reviewer (MHJ). As result, all 20 articles were retained and study quality was appraised using the Mixed Methods Appraisal Tool (MMAT) [18]. The MMAT is a critical appraisal tool that was developed to appraise studies included in systematic mixed study reviews. The methodology quality of five different types of studies (qualitative study, randomized controlled trial, non-randomized study, quantitative descriptive study and mixed methods study) can be appraised using this tool. The reporting of MMAT results is as follows: (i) five of the quality criteria met/ 100%; (ii) four of the quality criteria met/ 80%; (iii) three of the quality criteria met/ 60%; (iv) two of the quality criteria met/ 40%; and (v) one of the quality criteria met/ 20%.

### Data extraction and synthesis

MYJL, NA, ANA, and MHJ extracted the data independently using a standardized data extraction form which is organized using Microsoft Excel. The information collected in the form included: (1) author, (2) publication year, (3) references, (4) country, (5) study design, (6) study population, (7) sample size, (8) measure of diabetes risk perception, (9) statistical analysis, and (10) results. Due to the heterogeneity in the included studies in terms of study design, study population, and measure of diabetes risk perception, the findings of all included articles were synthesized using a narrative synthesis.

## Results

### Background of the eligible studies

A total of 20 studies were included in this systematic review. Table 2 shows the descriptive summary of the included studies. A total of seven studies were conducted in the United States, while two studies each were conducted in Australia, China, Finland, and Germany. The remaining five studies were conducted in Canada, Denmark, Iran, Japan, and Turkey. A total of 15 articles were published within the past five years (2018–2022), while the other five articles were published between 2014 and 2017. Of the included studies, 13 were cross-sectional studies, two each were randomized controlled trial, cohort, and mixed methods studies, and one was quasi-experiment with a control group. The detailed findings from the 20 studies included in this systematic review are presented along with their quality appraisal scoring in S1 Table.

**Table 2:** Descriptive summary of the included studies (n = 20).

Characteristics	Frequency	References
**Country**		
United States	7	[13, 19–24]
Australia	2	[25, 26]
China	2	[27, 28]
Finland	2	[29, 30]
Germany	2	[31, 32]
Canada	1	[14]
Denmark	1	[33]
Iran	1	[34]
Japan	1	[35]
Turkey	1	[36]
**Publication year**		
2022	3	[19, 20, 36]
2021	3	[25, 26, 29]
2020	3	[13, 14, 21]
2019	5	[22, 27, 28, 31, 34]
2018	1	[33]
2017	1	[32]
2016	3	[23, 24, 30]
2014	1	[35]
**Study design**		
Cross sectional	13	[13, 14, 19, 20, 23, 24, 26, 27, 30–33, 36]
Cohort	2	[28, 29]
Mixed methods	2	[21, 22]
Randomized controlled trial	2	[25, 35]
Quasi-experiment with a control group	1	[34]
**Study population (according to risk of diabetes)**		
Without apparent risk for diabetes	9	[13, 23, 24, 27, 29–33]
Had family history of diabetes	4	[14, 22, 35, 36]
Had current or previous diagnosis of gestational diabetes	6	[19, 20, 25, 26, 28, 34]
Had prediabetes	1	[21]
**Study population (according to gender)**		
Male-predominant	1	[35]
Female-predominant	14	[13, 19–23, 25–29, 33, 34, 36]
Equal proportion of male and female respondents	5	[14, 24, 30–32]
**Study population (according to race/ethnicity)**		
Non-Hispanic White	1	[13]
White and African American	1	[21]
Hispanic, non-Hispanic, Asian, non-Hispanic White, African American, and other race	1	[20]
Asian, Hispanic, Black, and White	1	[22]
US Latinos	2	[23, 24]
Asian	5	[14, 19, 27, 28, 35]
Not mentioned	9	[25, 26, 29–34, 36]
**Measure of diabetes risk perception**		
Standardized tool	6	[14, 19, 23, 27, 28, 34]
Investigator-developed items	13	[13, 20, 22, 24–26, 29–33, 35, 36]
Standardized tool and investigator-developed items	1	[21]

### Level of risk perception of diabetes

Nine out of the 20 studies assessed the level of risk perception of diabetes among individuals without apparent risk for diabetes (i.e., gestational diabetes mellitus, family history of diabetes, prediabetes). A total of four and six studies assessed the level of risk perception of diabetes among those with family history of diabetes and those who had current or previous diagnosis of gestational diabetes, respectively. The remaining one study assessed the risk perception of diabetes among individuals with prediabetes. The level of risk perception of diabetes was generally lower among those without apparent risk for diabetes. Studies found that 11.3% [27], 12.9% [32], 14.1% [31] and 30% [13] of individuals without apparent risk for diabetes perceived moderate/high risk of developing type 2 diabetes.

Among those who had family history of diabetes, 27.52% [22], 34.2% [14] and 62.7% [36] perceived high risk of developing type 2 diabetes. A randomized controlled trial conducted among individuals with family history of diabetes found that 34.6% of those in the intervention group and 40.6% of those in the control group perceived their risk of diabetes to be likely or very likely [35]. Studies found that 20% [20], 45% [25], 60.8% [28] and 72% [26] of women who were currently or previously diagnosed with gestational diabetes mellitus perceived high risk of type 2 diabetes. Other studies presented the scores of diabetes risk perception using mean and standard deviation without classifying the scores [19, 21, 23, 24, 29, 30, 33, 34].

Apart from that, the results of other domains of diabetes risk perception measured using the Risk Perception Survey for Developing Diabetes (RPS-DD) have been reported. For example, the mean (SD) scores of the worry domain (possible range 1 to 4) was 1.99 (0.55) [27] and 2.96 (0.80) [23] among those without apparent risk for diabetes, indicating slight to moderate concern about risk for developing diabetes. Similarly, the mean (SD) scores for the worry domain (possible range 2 to 8) before intervention was 4.70 (1.42), indicating moderate level of worry about developing diabetes among individuals with prediabetes [21].

The mean (SD) scores of the personal control domain of the RPS-DD has also been reported. Studies found that the mean (SD) scores of the personal control domain (possible range 1 to 4) was 3.05 (0.40) [27] and 3.34 (0.76) [23] among those without apparent risk for diabetes, indicating a tendency toward greater perceived personal control over the risk of development of diabetes. Similarly, the mean (SD) scores for the personal control domain (possible range 4 to 16) before intervention was 14.31 (1.60), indicating a high level of perceived personal control among individuals with prediabetes [21]. On the other hand, the mean (SD) prescores of the personal control domain (possible range 1 to 4) was 2.08 (0.45) and 2.36 (0.46) among intervention and control group of women with gestational diabetes, indicating lower level of perceived personal control [34].

The optimistic bias domain of diabetes risk perception was also studied. The mean (SD) scores of the optimistic bias domain (possible range 1 to 4) was 2.91 (0.56) [27] and 2.96 (0.92) [23] among those without apparent risk for diabetes, indicating a tendency to perceive lesser risk of developing diabetes compared to the others of the same age and sex. A study among women with gestational diabetes reported a comparable mean (SD) prescores of 3.07 (0.51) and 2.83 (0.52) among the intervention and control group [34]. In contrast, a study among adults with prediabetes revealed that the mean (SD) scores for the optimistic bias domain (possible range 2 to 8) before intervention was 3.33 (1.05), indicating their tendency to perceive higher risk of developing diabetes than their peers [21].

Three studies assessed the diabetes risk knowledge domain of diabetes risk perception. Guo et al. [27] and Joiner et al. [23] found that the mean (SD) scores for the diabetes risk knowledge domain (possible range 0 to 11) was 4.48 (2.23) and 4.36 (2.18), indicating limited knowledge about the risk factor for diabetes among those without apparent risk for diabetes. Similarly, Ghaderi et al. reported a comparable mean (SD) prescores of 4.22 (1.44) and 4.55 (1.50) among intervention and control group of women with gestational diabetes [34].

The results of the environmental health risk domain of diabetes risk perception were reported in a study conducted among individuals without apparent risk for diabetes [23]. This domain assesses the perception of risk to health of potential environmental health hazards (possible range 1 to 4). The mean (SD) score of 1.88 (0.79) indicated overall perceived slight risk to health across the potential environmental health hazards that are being assessed. The environmental hazard with the highest perception of risk mean score was secondary cigarette smoke (2.39), followed by household chemicals (2.19), air pollution (2.02) and pesticide (2.01), while several physical hazards such as extreme weather (1.81), driving/riding in an automobile (1.70) and violent crime (1.58) had lower perception of risk mean score.

### Measure of risk perception of diabetes

Of the 20 studies included in this systematic review, the only standardized tool used to assess the level of diabetes risk perception was the Risk Perception Survey for Developing Diabetes (RPS-DD) [14, 19, 21, 23, 27, 34]. One study used the complete version of the RPS-DD to measure risk perception of diabetes [34]. The complete version of the RPS-DD consists of 43 items with four domains, namely “personal control”, “optimistic bias”, diabetes risk knowledge”, and “benefits and barriers of preventive behaviours” (with Cronbach’s alphas ranging from 0.61 to 0.76). Joiner et al. used the complete, translated Spanish version of the RPS-DD, which consists of 43 items and includes six measures namely “personal disease risk scale”, “environment health risk scale”, personal control subscale”, “optimistic bias subscale”, worry subscale”, and diabetes risk knowledge test” (with Cronbach’s alphas ranging from 0.54 to 0.88) [23].

One study used a modified 33-item RPS-DD which evaluates “personal control”, “worry”, “optimistic bias”, “personal disease risk”, and “comparative environment risk” [14]. Guo et al. used a modified, translated Chinese version of the RPS-DD, which consists of 20 items and includes five subscales, namely “personal disease risk subscale”, “worry subscale”, “personal control subscale”, “optimistic bias subscale”, and “diabetes risk knowledge subscale” [27]. Another study conducted in China used the 12-item Chinese version of RPS-DD, which covers two domains namely “overall perceived risk” and “diabetes risk knowledge” [19]. A study conducted in the United States used the 8-item RPS-DD which includes the personal control, optimistic bias and worry subscales [21]. The only other standardized tool for assessing the level of risk perception of diabetes was the revised Chinese version of the Champion’s Health Belief Model Scale (RC-CHBMS) [28], while other remaining studies used between 1 to 13 items that were investigator-developed.

### Critical appraisal of the included studies

Of the 20 studies, one was given the score of 100%, nine were given the score of 80%, and 10 were given the score of 60%. The scores of MMAT for each included study are shown in S2 Appendix.

## Discussion

### The role of risk perception in diabetes

A study from the United States reported that the lifetime risk of diagnosed diabetes was 40.2% among men and 39.6% among women aged 20 years [37]. This represents a considerable proportion of the population as a potential target group for primary prevention. The risk perception of diabetes has been linked with the uptake of primary prevention strategies such as increasing physical activity and weight loss [12, 38]. However, despite the available evidence regarding the risk factors of diabetes [39], a substantial proportions of apparently high-risk individuals of the included studies did not link their risk factors with an increased perceived risk. This may be explained by people’s tendency to maintain a favourable impression of their health status and health behaviours [40].

Studies involving individuals without apparent risk for diabetes reported an overall lower level of risk perception of diabetes compared with studies involving respondents with family history of diabetes or history of gestational diabetes mellitus. Such level of risk perception is congruent with the results of the worry domain of diabetes risk perception. This finding is also consistent with earlier research which found that even though both the low-risk and high-risk individuals had relatively low risk perception of diabetes, the mean perceived risk was significantly lower for the low-risk compared to the high-risk individuals [41].

The lower level of risk perception of diabetes among individuals without apparent risk for diabetes may have been influenced by their greater perceived personal control over the risk of diabetes as well as their optimistic bias. Previous studies found that optimistic bias is highlighted when the risk are perceived to be controllable [42, 43]. The well-known fact that diabetes is largely preventable due to modifiable behaviours (smoking, physical inactivity, unhealthy diet) may have contributed to the greater perceived personal control over the risk of diabetes [44]. On the other hand, even though individuals with prediabetes reported high level of perceived personal control, they showed less optimistic bias [21]. Individuals with apparent risk for diabetes may see the development of diabetes as inevitable and therefore show low correspondence between control and risk judgements [43].

This review found that individuals without apparent risk for diabetes and women with gestational diabetes had limited knowledge about the risk factor for diabetes. Other study reported contrary results that women with history of gestational diabetes had greater knowledge about the risk factor for diabetes [45]. However, our finding is in line with previous studies which demonstrated low level of knowledge about diabetes and its risk factors among general population with and without risk for diabetes [46, 47]. The lack of knowledge of risk factor of diabetes may also explain the low level of diabetes risk perception found in this review [48]. Thus, the risk perception of diabetes may be increased through the increase of knowledge to motivate preventive health behaviours.

The generalizability of findings is dependent on obtaining a representative sample of the broader population [49]. Most of the included studies involved predominantly or entirely female participants and therefore the external validity of the findings is limited. For example, the effect of sampling on the validity of findings can be seen in a study conducted among Filipino, Korean, and Latino Americans in the United States in which women were more likely to perceive risk of diabetes than men [50]. Thus, more studies that incorporate men are needed in the future.

### The measurement of risk perception of diabetes

There is lack of consistency across these studies which make it difficult to compare and draw conclusions from the findings. The measures used to calculate the level of risk perception of diabetes were widely varied across these studies. We also observed an overall lack of statistics supporting the reliability and validity of the tool used to measure the risk perception of diabetes. Even though several studies have used a previously validated tool to measure risk perception of diabetes, not all reported the psychometrics of the tool. The reporting of psychometric estimates is important to ensure that the scales and subscales measure the construct consistently and accurately [49].

Regarding the tools used for measuring the level of risk perception of diabetes, Ghaderi et al. evaluated the face and content validity of the RPS-DD, and reported the Cronbach’s alphas which ranged from 0.61 for the “optimistic bias” domain to 0.76 for the “diabetes risk knowledge” domain [34]. Another study by Joiner et al. evaluated the RPS-DD through field testing, and reported the Cronbach’s alphas which ranged between 0.54 and 0.88 [23]. Nevertheless, the lack of composite score for the RPS-DD and the wide variations in tools used for measuring the risk perception of diabetes weaken the authors’ ability to compare the level of risk perception of diabetes across studies.

### The role of environmental factor: A review of the literature

The risk judgements about environmental health hazards provide a broader context for understanding personal risk of diabetes [51]. People commonly have complicated lives, having to cope with various health problems in the context of potential environmental health hazards. Hence, exploring the layers of this complexity may help to better understand the risk perception of diabetes and facilitate the formulation of better preventive strategies. The perception of slight risk to health across the environmental health hazard found in this review is in line with earlier studies in the United States involving nondiabetic healthcare personnel [52, 53] and adults with prediabetes [54]. Even though the perception of risk to health of environmental exposure is low on average and may seem insignificant, people might be worried that the risk falls disproportionally on the vulnerable groups and thus call for action [55].

As identified in this review, secondary cigarette smoke had the highest perception of risk mean score. This is in line with expectation, as it is well known that secondhand smoke is associated with increased risk of type 2 diabetes [56]. Similarly, the fact that long-term exposure to air pollution is associated with increased risk of diabetes may have contributed to the high perception of risk mean score for air pollution [57]. On the other hand, violent crime had among the lowest perception of risk mean score. This is explainable as people may not readily associate violent crime with diabetes due to its indirect effect on the occurrence of diabetes. For example, crime and unsafe neighbourhood may incite isolation and fear, causing physical inactivity which can increase a person’s risk of developing diabetes. Unsafe neighbourhood may also lead to stress, which can damage the immune and body systems and accelerate the development of chronic diseases such as diabetes [58].

Exposure to the environmental factors should be considered when assessing the risk perception of diabetes. Evidence suggested that risk perception is not only influenced by beliefs, attitudes, wider social or cultural values, and past experiences [55], but is also closely related to an individual’s experience in his/her geographical and climatic environment [59]. The results of a grounded theory also suggested that risk perception incorporates a complex psychological understanding on the formation of diabetes risk, which takes into consideration the social, cultural, and community-based environments. For instance, the environment in the South Asian community actively encourage the use of shisha, and quitting shisha was also not considered as a modifiable risk factor for diabetes. This may not only have a direct effect on the prevalence of diabetes, but may also influences the risk perception of diabetes [60].

Despite the important role of environment factors in risk perception of diabetes, it is rarely examined in studies. Even though the environmental health risk domain was included in the RPS-DD, many studies did not include this domain while assessing the risk perception of diabetes using this tool [19, 27, 34]. This provides a reasonable basis for further study in this area to obtain better evidence to support the development of diabetes preventive interventions that aimed at increasing the risk perception of diabetes.

### Strengths and limitations

This systematic review is not without limitations. According to the MMAT results, the mixed methods studies lacked information about accounting for confounders, sampling strategy, participant representativeness and risk of nonresponse bias. For randomized controlled trials, the lack of complete outcome data and description of blinding were the underlying reasons for lower score. For other quantitative studies, low scores were associated with insufficient explanations about sample representativeness and risk of nonresponse bias. The use of study-specific and non-validated measures in the included studies weakens the authors’ ability to compare across studies. Improving the measurement of risk perception of diabetes could help standardize the diabetes risk perception literature.

The role of language and publication bias must be considered as we included only English-language studies published in peer-reviewed journals. Another limitation is the exclusion of dimensions of risk perception such as optimistic bias as search terms. Nevertheless, even studies that examined one dimension of risk perception can add value to the overall knowledge of risk perception of diabetes. Additionally, this review followed the PRISMA 2020 statement for the reporting of systematic reviews to ensure reporting quality.

## Conclusion

This review found an overall low level of diabetes risk perception, particularly in those without apparent risk for diabetes. Most studies reported slight to moderate concern about risk for developing diabetes (worry domain), greater perceived personal control over the risk of development of diabetes (personal control domain), perception of lower risk of developing diabetes when compared with peers (optimistic bias domain) and limited knowledge about the risk factor for diabetes (diabetes risk knowledge domain).

Risk perception has been linked to health behaviours required to prevent diabetes. However, the overall low risk perception of diabetes is worrying considering the severity of the disease and the preventive measures available. This calls for urgent need of public health interventions that can increase the risk perception of diabetes. Future research on risk perception of diabetes should focus on both men and women, as well as on strategies to increase the risk perception of diabetes to promote preventive health behaviours.

Researchers use various measures to assess risk perception of diabetes. However, it is imperative that researchers keep in mind the importance of ensuring validity and reliability of measures used in future research. With a better understanding of risk perception of diabetes, diabetes preventive interventions can be developed or enhanced accordingly. The influence of environmental factors on risk perception of diabetes indicates that diabetes preventive interventions targeting environmental factors may be effective in increasing the risk perception of diabetes. Nevertheless, more studies are required to better understand the role of environment factors in risk perception of diabetes.

## Supporting information

S1 Appendix PRISMA 2020 checklist. (DOCX)

S2 Appendix Critical appraisal of selected studies using MMAT. (DOCX)

S1 Table Data. (XLSX)
