# Supplementary material for: A systematic review on the level of risk perception of diabetes mellitus: The role of environmental factor
Source: PLoS One. 2024 Jul 30;19(7):e0308152. doi: 10.1371/journal.pone.0308152 (PMC11288419; doi:10.1371/journal.pone.0308152)
Supplement: S2 Appendix — (DOCX) [file pone.0308152.s002.docx]

**Critical appraisal of selected studies using MMAT.**

| **Category of study designs** | **Methodological quality criteria** |
| --- | --- |
| 1. Qualitative | 1.1 Is the qualitative approach appropriate to answer the research question? |
|  | 1.2 Are the qualitative data collection methods adequate to address the research question? |
|  | 1.3 Are the findings adequately derived from the data? |
|  | 1.4 Is the interpretation of results sufficiently substantiated by data? |
|  | 1.5 Is there coherence between qualitative data sources, collection, analysis and interpretation? |
| 2. Quantitative randomized controlled trials | 2.1 Is randomization appropriately performed? |
|  | 2.2 Are the groups comparable at baseline? |
|  | 2.3 Are there complete outcome data? |
|  | 2.4 Are outcome assessors blinded to the intervention provided? |
|  | 2.5 Did the participants adhere to the assigned intervention? |
| 3. Quantitative non-randomized | 3.1 Are the participants representative of the target population? |
|  | 3.2 Are measurements appropriate regarding both the outcome and intervention (or exposure)? |
|  | 3.3 Are there complete outcome data? |
|  | 3.4 Are the confounders accounted for in the design and analysis? |
|  | 3.5 During the study period, is the intervention administered (or exposure occurred) as intended? |
| 4. Quantitative descriptive | 4.1 Is the sampling strategy relevant to address the research question? |
|  | 4.2 Is the sample representative of the target population? |
|  | 4.3 Are the measurements appropriate? |
|  | 4.4 Is the risk of nonresponse bias low? |
|  | 4.5 Is the statistical analysis appropriate to answer the research question? |
| 5. Mixed methods | 5.1 Is there an adequate rationale for using a mixed methods design to address the research question? |
|  | 5.2 Are the different components of the study effectively integrated to answer the research question? |
|  | 5.3 Are the outputs of the integration of qualitative and quantitative components adequately interpreted? |
|  | 5.4 Are divergences and inconsistencies between quantitative and qualitative results adequately addressed? |
|  | 5.5 Do the different components of the study adhere to the quality criteria of each tradition of the methods involved? |

| **No.** | **Author, Year** | **1.1** | **1.2** | **1.3** | **1.4** | **1.5** | **2.1** | **2.2** | **2.3** | **2.4** | **2.5** | **3.1** | **3.2** | **3.3** | **3.4** | **3.5** | **4.1** | **4.2** | **4.3** | **4.4** | **4.5** | **5.1** | **5.2** | **5.3** | **5.4** | **5.5** | **Quality**  **(%)** |
| --- | --- | --- | --- | --- | --- | --- | --- | --- | --- | --- | --- | --- | --- | --- | --- | --- | --- | --- | --- | --- | --- | --- | --- | --- | --- | --- | --- |
| 1 | Huang et al. 2022 |  |  |  |  |  |  |  |  |  |  |  |  |  |  |  | Yes | Can’t tell | Yes | Can’t tell | Yes |  |  |  |  |  | 60 |
| 2 | Vu et al. 2022 |  |  |  |  |  |  |  |  |  |  |  |  |  |  |  | Yes | Can’t tell | Yes | Can’t tell | Yes |  |  |  |  |  | 60 |
| 3 | Yilmaz et al. 2022 |  |  |  |  |  |  |  |  |  |  |  |  |  |  |  | Yes | Yes | Yes | Can’t tell | Yes |  |  |  |  |  | 80 |
| 4 | Gray et al. 2021 |  |  |  |  |  | Yes | Yes | No | Can’t tell | Yes |  |  |  |  |  |  |  |  |  |  |  |  |  |  |  | 60 |
| 5 | O'reilly et al. 2021 |  |  |  |  |  |  |  |  |  |  |  |  |  |  |  | Yes | Can’t tell | Yes | No | Yes |  |  |  |  |  | 60 |
| 6 | Vornanen et al. 2021 |  |  |  |  |  |  |  |  |  |  |  |  |  |  |  | Yes | Yes | Yes | Yes | Yes |  |  |  |  |  | 100 |
| 7 | Antwi et al. 2020 |  |  |  |  |  |  |  |  |  |  |  |  |  |  |  | Yes | Can’t tell | Yes | No | Yes |  |  |  |  |  | 60 |
| 8 | Koipuram et al. 2020 |  |  |  |  |  |  |  |  |  |  |  |  |  |  |  | Yes | Yes | Yes | Can’t tell | Yes |  |  |  |  |  | 80 |
| 9 | Yost et al. 2020 | Yes | Yes | Yes | Yes | Yes |  |  |  |  |  | Can’t tell | Yes | Yes | No | Yes |  |  |  |  |  | Yes | Yes | Yes | Yes | Yes | 60 |
| 10 | Daack‐Hirsch et al. 2019 | Yes | Yes | Yes | Yes | Yes |  |  |  |  |  |  |  |  |  |  | Can’t tell | Yes | Yes | Can’t tell | Yes | Yes | Yes | Yes | Yes | Yes | 60 |
| 11 | Ghaderi et al. 2019 |  |  |  |  |  |  |  |  |  |  | Yes | Yes | No | Yes | Yes |  |  |  |  |  |  |  |  |  |  | 80 |
| 12 | Guo et al. 2019 |  |  |  |  |  |  |  |  |  |  |  |  |  |  |  | Can’t tell | Yes | Yes | Yes | Yes |  |  |  |  |  | 80 |
| 13 | Heidemann et al. 2019 |  |  |  |  |  |  |  |  |  |  |  |  |  |  |  | Yes | Yes | Yes | Can’t tell | Yes |  |  |  |  |  | 80 |
| 14 | Liu et al. 2019 |  |  |  |  |  |  |  |  |  |  |  |  |  |  |  | Yes | Can’t tell | Yes | Yes | Yes |  |  |  |  |  | 80 |
| 15 | Skøt et al. 2018 |  |  |  |  |  |  |  |  |  |  |  |  |  |  |  | Yes | Can’t tell | Yes | No | Yes |  |  |  |  |  | 60 |
| 16 | Kowall et al. 2017 |  |  |  |  |  |  |  |  |  |  |  |  |  |  |  | Yes | Yes | Yes | No | Yes |  |  |  |  |  | 80 |
| 17 | Basilio et al. 2016 |  |  |  |  |  |  |  |  |  |  |  |  |  |  |  | Yes | Can’t tell | Yes | Yes | Yes |  |  |  |  |  | 80 |
| 18 | Joiner et al. 2016 |  |  |  |  |  |  |  |  |  |  |  |  |  |  |  | Yes | Can’t tell | Yes | Can’t tell | Yes |  |  |  |  |  | 60 |
| 19 | Vornanen et al. 2016 |  |  |  |  |  |  |  |  |  |  |  |  |  |  |  | Yes | Yes | Yes | No | Yes |  |  |  |  |  | 80 |
| 20 | Nishigaki et al. 2014 |  |  |  |  |  | Yes | Yes | No | Can’t tell | Yes |  |  |  |  |  |  |  |  |  |  |  |  |  |  |  | 60 |
